# Editorial: Chlorophyll fluorescence analysis in biotic and abiotic stress, volume II

**DOI:** 10.3389/fpls.2022.1066865

**Published:** 2022-11-14

**Authors:** Michael Moustakas, Lucia Guidi, Angeles Calatayud

**Affiliations:** ^1^ Department of Botany, School of Biology, Aristotle University of Thessaloniki, Thessaloniki, Greece; ^2^ Department of Agriculture, Food and Environment, University of Pisa, Pisa, Italy; ^3^ Instituto Valenciano de Investigaciones Agrarias, Centro de Citricultura y Producción Vegetal, Departamento de Horticultura, Valencia, Spain

**Keywords:** photochemical efficiency, reactive oxygen species, environmental stress, oxidative stress, photosystem II, non-photochemical quenching (NPQ), energy partitioning

Chlorophyll *a* fluorescence emission results from absorbed light energy that is not dissipated as heat or not used for photosynthetic reactions in plants. Photosynthesis is allocated into two distinct parts, the light reactions and the carbon dioxide (CO_2_) fixation. In the light reactions, light energy is utilized to generate an oxidized protein complex capable of extracting electrons from water at photosystem II (PSII), and at the same time re-energizing the extracted electron to reduce NADP^+^ at photosystem I (PSI). These ‘light harvesting’ reactions result in the formation of ATP and reducing power (reduced ferredoxin and NADPH), and subsequent CO_2_ fixation through the Calvin–Benson–Bassham cycle. Chlorophyll *a* fluorescence analysis can determine the amount of absorbed light energy that is directed to photochemistry and estimates photosynthetic efficiency under biotic or abiotic stresses (Moustakas et al., 2021; Moustakas, 2022). Chlorophyll *a* fluorescence signals can be interpreted in terms of photosynthetic activity to obtain information about the state of the photosynthetic apparatus and especially of photosystem II (PSII) (Murchie and Lawson, 2013; Moustakas et al., 2021). Measurements of chlorophyll *a* fluorescence have been extensively used to probe the function of the photosynthetic machinery and for screening different crops for plant tolerance to numerous stresses, and nutritional requirements (Guidi and Calatayud, 2014; Kalaji et al., 2016; Sperdouli et al., 2021; Moustakas et al., 2022a). The use of the pulse amplitude modulation (PAM) method can principally calculate the amount of absorbed light energy that is directed to PSII for photochemistry, which is dissipated as heat through the non-photochemical quenching (NPQ) mechanism or dissipated by less well characterized non-radiative fluorescence processes, that are marked as Φ*_PSII_*, Φ*_NPQ_*, and Φ*_NO_*, respectively, with the sum of them to be equal to one (Kramer et al., 2004).

In the present work, we summarize the articles included in this special issue, updating readers on the subject and discussing current applications of chlorophyll fluorescence analysis. Chlorophyll fluorescence analysis has been implicated in many studies with the addition of external substances (e.g., proline, carbohydrates, salicylic acid, melatonin, etc.) to enhance photosynthetic efficiency or ameliorate stress effects in plants (Moustakas et al., 2022b). Melatonin application improved photosynthetic activity in maize seedlings under drought stress through a higher photochemical activity mediated by the activation of antioxidative defense (Huang et al., 2019). The alleviation of water stress effects by melatonin were higher when melatonin was applied to the roots compared with a foliar spray, indicating a melatonin signal from roots to leaves (Huang et al., 2019). In wheat, 25 μM melatonin application alleviated the decline in photosynthetic efficiency under water stress, by effective protection of the photosynthetic apparatus, through the regulation of PSII proteins and the reversible phosphorylation of the thylakoid proteins (Lin et al.). In cucumber seedlings, melatonin promoted chilling tolerance through the activation of antioxidant enzymes and the induction of carbon assimilation genes as well as by key PSI-PSII-related genes, which alleviated damage to the photosynthetic apparatus and decreased oxidative damage under chilling stress (Zhang et al.).

Light is essential for photosynthesis, but excessive light can cause oxidative damage to the photosynthetic apparatus by producing reactive oxygen species (Takahashi and Badger, 2011). In this way, plants have evolved an array of photoprotection mechanisms to alleviate the harmful effects of high light intensity, and, among them, the most rapid and efficient mechanism is represented by non-photochemical quenching (NPQ), which is most effective in the joint presence of the PsbS (PSII subunit S) protein and the xanthophyll cycle (Welc et al., 2021; Ruban and Wilson, 2021). Lou et al. found that high light intensity increased NPQ and stimulated the de-epoxidation of violaxanthin cycle components in *Phyllostachys edulis*, while the treatment of plants with dithiothreitol, a violaxanthin de-epoxidase (VDE) inhibitor, induced a reduction in NPQ ability, confirming the strict relationship between violaxanthin cycle and photoprotection mechanism of bamboo. The authors evidenced also, that extreme temperatures (4 and 42°C) and drought stress upregulated the expression of *PeVDE* in bamboo leaves. The *PeVDE* gene of moso bamboo (*Phyllostachys edulis*) is expressed primarily in leaves and the encoded protein has been shown to convert violaxanthin to zeaxanthin. Transgenic plants overexpressing *PeVDE* showed an enhanced photoprotection ability, higher NPQ capacity, and a slower decline in the maximum quantum yield of photosystem II (F_v_/F_m_) under high light intensity as compared with wild-type (Col-0) plants. Lou et al. concluded that the *PeVDE* gene has a positive role in the response to high light intensity in bamboo plants improving their photoprotection ability through the violaxanthin cycle and NPQ.

In the context of climate change, heat stress is one of the most important constraints limiting plant production and quality. For wheat, an essential heat-sensitive cereal crop for the human diet, finding a research strategy to overcome heat stress is important (Lipec et al., 2013). Fei et al. report on a study that aimed to characterize whether delayed sowing could promote heat stress tolerance in winter wheat compared to “normal” sowing. By using an agronomic, photosynthetic, and proteomic analysis, the study evidenced that delayed sowing improved tiller survival percentage, maintained higher photosynthetic capacity mediated by upregulation of the photosynthesis related proteins (PsbH and PsbR), and increased oxygen radical scavenging capacity. These results reveal that delaying sowing is an effective agronomy technique for confering heat tolerance in wheat.

Overall, from the significant articles presented in this Research Topic, it was concluded that chlorophyll *a* fluorescence analysis is a widely used method that is easy, quick, cheap, non-invasive, and highly sensitive in determining photosynthetic efficiency and detecting the impact of stresses on plants. The contributions included in this e-book are useful for scientists working on the subject, outlining and explaining recent advances on this topic.

## Author contributions

All authors listed, have made a substantial, direct, and intellectual contribution to the work, and approved it for publication.

## Conflict of interest

The authors declare that the research was conducted in the absence of any commercial or financial relationships that could be construed as a potential conflict of interest.

## Publisher’s note

All claims expressed in this article are solely those of the authors and do not necessarily represent those of their affiliated organizations, or those of the publisher, the editors and the reviewers. Any product that may be evaluated in this article, or claim that may be made by its manufacturer, is not guaranteed or endorsed by the publisher.
